# Supplementary material for: Divergent density feedback control of migratory predator recovery following sex‐biased perturbations
Source: Ecol Evol. 2020 Apr 8;10(9):3954–67. doi: 10.1002/ece3.6153 (PMC7244814; doi:10.1002/ece3.6153)
Supplement: Supplementary file 1 — Table S1‐S2 [file ECE3-10-3954-s001.docx]

**Supporting Information**

Table S1. Summary of exposure period (27 years) model outputs (mean ± sd) from the shovelnose sturgeon spatially explicit individual-based model simulations for energetic (reduced energy allocation to female gonad development) and demographic (pre-recruit sex ratio skewness) perturbation scenarios. Vt_low, Vt_mid, and Vt_high indicate vitellogenin reduction in adult females exposed to low-, mid-, and high- level synthetic androgen. SS_low, SS_mid, and SS_high indicate skewed sex ratio in larvae exposed to low-, mid-, and high- level synthetic androgen. Numbers indicates mean values computed across years.

| model output | baseline | Vt_low | Vt_mid | Vt_high | SS_low | SS_mid | SS_high | Vt + SS_low | Vt + SS_mid | Vt + SS_high |
| --- | --- | --- | --- | --- | --- | --- | --- | --- | --- | --- |
| **adult female** |  |  |  |  |  |  |  |  |  |  |
| sex ratio (proportion of female) | 0.31±0.15 | 0.32±0.13 | 0.32±0.13 | 0.32±0.13 | 0.29±0.14 | 0.27±0.13 | 0.24±0.11 | 0.31±0.12 | 0.27±0.11 | 0.27±0.12 |
| mean age of adult female | 6.88±0.56 | 6.86±0.52 | 6.85±0.52 | 6.86±0.52 | 6.90±0.57 | 6.92±0.56 | 6.89±0.55 | 6.88±0.53 | 6.87±0.54 | 6.86±0.52 |
| reproductive female abundance | 3119.0±2260.5 | 2819.4±1979.2 | 2505.4±1782.5 | 2002.4±1550.5 | 2643.6±2023.5 | 2532.6±1908.1 | 2475.2±1836.0 | 2138.0±1596.5 | 2092.3±1542.9 | 1648.4±1407.2 |
| mean relative storage mass of reproductive female | 0.24±0.04 | 0.24±0.04 | 0.24±0.04 | 0.24±0.04 | 0.24±0.04 | 0.24±0.04 | 0.24±0.04 | 0.24±0.04 | 0.24±0.04 | 0.24±0.03 |
| mean relative storage mass of non-reproductive female | 0.25±0.02 | 0.24±0.05 | 0.24±0.02 | 0.25±0.02 | 0.24±0.04 | 0.25±0.03 | 0.24±0.04 | 0.24±0.03 | 0.24±0.02 | 0.24±0.03 |
| mean relative gonad mass of reproductive female | 0.10±0.03 | 0.10±0.03 | 0.10±0.03 | 0.10±0.03 | 0.10±0.03 | 0.10±0.03 | 0.10±0.03 | 0.10±0.03 | 0.10±0.03 | 0.10±0.03 |
| number of spawner | 701.6±708.3 | 714.3±670.4 | 680.1±.670.7 | 467.1±572.5 | 604.4±646.7 | 600.0±653.5 | 577.6±645.5 | 500.8±593.8 | 480.0±577.6 | 406.2±558.2 |
| mean spawning location (rkm) | 103.9±.12.9 | 103.7±.13.2 | 100.1±13.9 | 99.0±.13.9 | 103.4±.11.3 | 103.9±15.7 | 104.0±.15.2 | 102.2±.17.1 | 100.4±.15.5 | 124.8±.14.0 |
| mean spawning time (day of year) | 117.6±.12.1 | 116.0±.10.6 | 115.3±10.6 | 113.7±10.6 | 117.3±.14.3 | 118.5±12.6 | 118.5±.11.6 | 115.6±.11.0 | 114.1±.12.7 | 113.5±.11.5 |
| mean egg number per female | 33369.5±2697.1 | 33622.5±2570.8 | 33965.5±2571.0 | 34667.3±2882.0 | 32899.3±3189.0 | 33074.0±3232.3 | 33023.7±3173.8 | 33551.3±3217.0 | 33815.4±3145.3 | 34326.9±3423.3 |
| mean spawning interval (years) | 2.81±0.52 | 2.91±0.53 | 2.88±0.54 | 3.01±0.55 | 2.91±0.66 | 2.89±0.55 | 2.77±0.54 | 3.04±0.69 | 3.00±0.55 | 3.99±0.55 |
| **early life stages** |  |  |  |  |  |  |  |  |  |  |
| total egg number (millions) | 22.7±22.1 | 20.1±20.6 | 18.1±18.9 | 15.2±16.9 | 19.8±20.1 | 19.5±20.1 | 22.5±19.9 | 16.2±17.6 | 15.6±17.2 | 17.7±18.4. |
| mean larval settlement location (rkm) | 100.2±20.9 | 97.7±16.6 | 92.7±15.4 | 92.4±17.3 | 100.3±22.4 | 97.9±21.1 | 99.1±19.2 | 97.4±15.9 | 99.8±17.8 | 92.8±17.9 |
| mean first-year survival rate (%) | 0.11±0.22 | 0.11±0.18 | 0.085±0.14 | 0.074±0.12 | 0.063±0.097 | 0.054±0.095 | 0.057±0.10 | 0.057±0.092 | 0.069±0.12 | 0.041±0.083 |
| total age 0 recruit number | 8323.5±16732.8 | 7750.3±14896.6 | 5782.8±11599.4 | 3479.3±7535.8 | 6579.9±12886.2 | 5812.1±11200.5 | 8050.7±15081.8 | 3463.1±7271.0 | 4192.3±9077.9 | 3656.4±7152.8 |
| age 0 recruit sex ratio (proportion of female) | 0.57±0.22 | 0.56±0.19 | 0.48±0.13 | 0.52±0.22 | 0.47±0.20 | 0.44±0.18 | 0.38±0.19 | 0.48±0.23 | 0.55±0.17 | 0.52±0.25 |

Table S2. Summary of recovery period (30 years) model outputs (mean ± sd) from the shovelnose sturgeon spatially explicit individual-based model simulations for energetic (reduced energy allocation to female gonad development) and demographic (skewed sex ratio) perturbation scenarios. Vt_low, Vt_mid, and Vt_high indicate vitellogenin reduction in adult females exposed to low-, mid-, and high- level synthetic androgen. SS_low, SS_mid, and SS_high indicate skewed sex ratio in larvae exposed to low-, mid-, and high- level synthetic androgen.

| model output | baseline | Vt_low | Vt_mid | Vt_high | SS_low | SS_mid | SS_high | Vt + SS_low | Vt + SS_mid | Vt + SS_high |
| --- | --- | --- | --- | --- | --- | --- | --- | --- | --- | --- |
| **adult female** |  |  |  |  |  |  |  |  |  |  |
| sex ratio (proportion of female) | 0.31±0.11 | 0.31±0.09 | 0.31±0.09 | 0.31±0.08 | 0.31±0.11 | 0.29±0.09 | 0.28±0.13 | 0.30±0.09 | 0.28±0.09 | 0.29±0.11 |
| mean age of adult female | 6.69±0.17 | 6.71±0.23 | 6.67±0.21 | 6.51±0.64 | 6.70±0.31 | 6.67±0.24 | 6.80±0.47 | 6.64±0.28 | 6.67±0.20 | 6.55±0.13 |
| reproductive female abundance | 2282.5±1024.0 | 2958.4±1212.7 | 2044.6±831.4 | 837.8±426.5 | 2087.0±1034.9 | 822.3±404.0 | 1133.6±1052.6 | 1458.2±713.4 | 1867.9±1183.2 | 1022.3±516.7 |
| mean relative storage mass of reproductive female | 0.25±0.02 | 0.25±0.02 | 0.25±0.02 | 0.26±0.02 | 0.25±0.02 | 0.25±0.02 | 0.25±0.02 | 0.25±0.01 | 0.25±0.02 | 0.26±0.01 |
| mean relative storage mass of non-reproductive female | 0.24±0.01 | 0.24±0.01 | 0.24±0.01 | 0.23±0.02 | 0.23±0.04 | 0.22±0.04 | 0.22±0.04 | 0.23±0.02 | 0.23±0.02 | 0.23±0.03 |
| mean relative gonad mass of reproductive female | 0.09±0.01 | 0.09±0.01 | 0.09±0.01 | 0.09±0.01 | 0.09±0.02 | 0.09±0.01 | 0.09±0.01 | 0.09±0.01 | 0.09±0.01 | 0.09±0.01 |
| number of spawner | 436.0±356.5 | 504.6±459.0 | 355.9±335.3 | 145.9±122.4 | 357.5±258.6 | 137.6±114.4 | 209.9±291.4 | 256.8±172.1 | 346.8±341.3 | 178.4±133.2 |
| mean spawning location (rkm) | 102.1±.6.3 | 100.6±6.2 | 102.6±.7.9 | 102.7±.6.8 | 103.4±.11.3 | 104.1±7.8 | 102.3±.7.0 | 104.0±6.8 | 101.8±.7.8 | 102.6±7.3 |
| mean spawning time (day of year) | 119.0±.5.6 | 121.5±6.4 | 120.0±7.6 | 120.7±6.0 | 118.8±9.6 | 119.7±6.6 | 118.3±8.2 | 114.8±.6.4 | 119.4±7.0 | 119.0±8.4 |
| mean egg number per female | 33886.9±1414.8 | 33499.4±1177.1 | 33925.7±1221.6 | 33833.5±1285.7 | 34562.2±1880.3 | 34199.4±1487.3 | 34551.2±1463.8 | 34768.0±1365.8 | 34104.0±1659.9 | 34905.2±1858.5 |
| mean spawning interval (years) | 3.18±0.36 | 3.08±0.43 | 3.24±0.42 | 3.21±0.47 | 3.27±0.38 | 3.24±0.36 | 3.09±0.37 | 3.31±0.31 | 3.18±0.36 | 3.22±0.40 |
| **early life stages** |  |  |  |  |  |  |  |  |  |  |
| total egg number (millions) | 14.7±12.1 | 17.2±11.9 | 12.1±9.03 | 4.87±3.99 | 12.1±8.48 | 4.71±3.94 | 8.39±12.4 | 8.85±5.88 | 11.8±11.6 | 5. 90±5.35 |
| mean larval settlement location (rkm) | 96.8±8.9 | 95.9±10.0 | 97.6±10.9 | 94.6±9.5 | 97.7±10.8 | 99.0±11.2 | 98.2±13.7 | 99.1±10.3 | 97.6±9.0 | 95.9±10.8 |
| mean first-year survival rate (%) | 0.11±0.19 | 0.13±0.17 | 0.11±0.15 | 0.13±0.17 | 0.076±0.011 | 0.13±0.22 | 0.076±0.15 | 0.11±0.18 | 0.28±0.99 | 0.20±0.24 |
| total age 0 recruit number | 9340.3± 15274.4 | 11045.1±16848.3 | 6584.9±10952.2 | 3191.5±4420.9 | 5497.5±10621.3 | 2761.4±4475.5 | 2030.62±4863.7 | 3920.8±5213.8 | 7666.8±14721.8 | 4188.1±5734.9 |
| age 0 recruit sex ratio (proportion of female) | 0.56±0.16 | 0.52±0.10 | 0.51±0.11 | 0.51±0.16 | 0.51±0.20 | 0.53±0.18 | 0.52±0.22 | 042±0.17 | 0.47±0.17 | 0.53±0.23 |

REFERENCES FOR APPENDIX S1

Goto, D., Hamel, M.J., Hammen, J.J., Rugg, M.L., Pegg, M.A. & Forbes, V.E. (2015) Spatiotemporal variation in flow-dependent recruitment of long-lived riverine fish: Model development and evaluation. *Ecological modelling,* **296,** 79-92.

Wildhaber, M.L., Papoulias, D.M., DeLonay, A.J., Tillitt, D.E., Bryan, J.L. & Annis, M.L. (2007) Physical and hormonal examination of Missouri River shovelnose sturgeon reproductive stage: a reference guide. *Journal of Applied Ichthyology,* **23,** 382-401.S
