# Supplementary material for: Divergent density feedback control of migratory predator recovery following sex‐biased perturbations
Source: Ecol Evol. 2020 Apr 8;10(9):3954–67. doi: 10.1002/ece3.6153 (PMC7244814; doi:10.1002/ece3.6153)
Supplement: Supplementary file 2 — Figure S1 [file ECE3-10-3954-s002.docx]

**
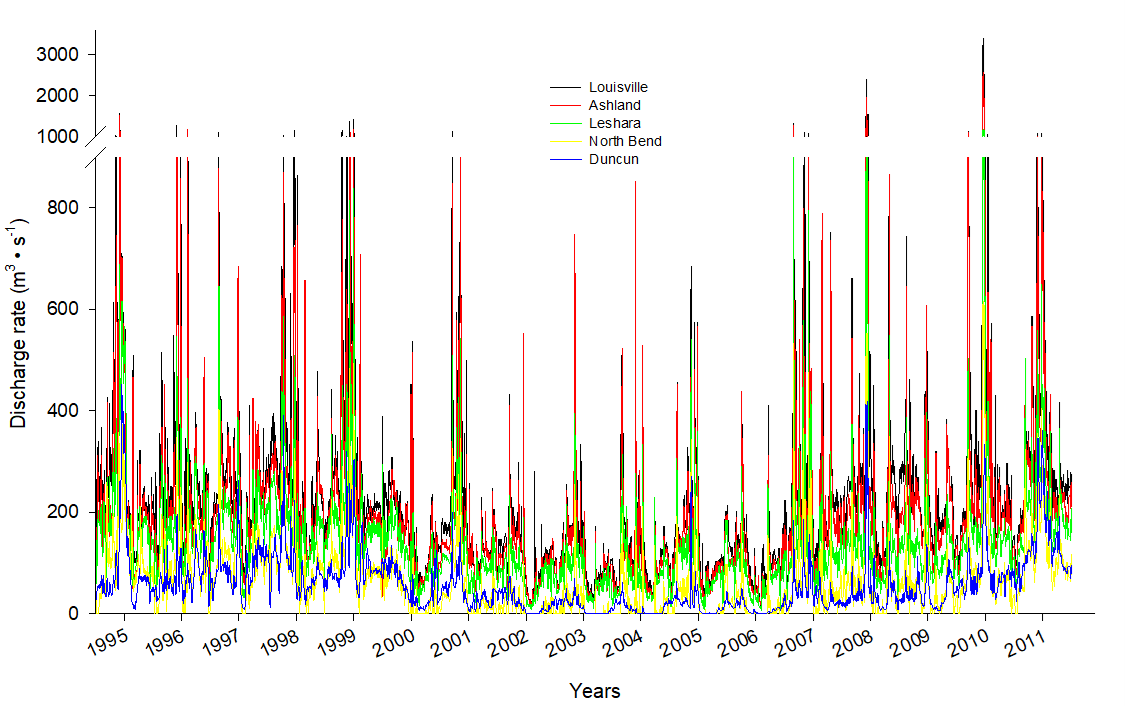
Figure S1**. Daily mean river discharge rates measured at five USGS gaging stations (Duncan– station #06774000, North Bend–#06796000, Leshara–#06796500, Ashland–#06801000, and Louisville–#06805500) during 1995–2011.
