# Supplementary material for: Divergent density feedback control of migratory predator recovery following sex‐biased perturbations
Source: Ecol Evol. 2020 Apr 8;10(9):3954–67. doi: 10.1002/ece3.6153 (PMC7244814; doi:10.1002/ece3.6153)
Supplement: Supplementary file 3 — Figure S2 [file ECE3-10-3954-s003.docx]

**Figure S2**. a) Relationship between energy allocation to female gonad development and vitellogenin computed from field estimates of vitellogenin concentrations and gonadosomatic index in pre-spawning female shovelnose sturgeon reported in Wildhaber et al. (2007); and b) simulation scenarios tested for the maternal investment in egg production (energetic perturbation). In a, Stage I, II, III, IV, and V represent immature, developing, vitellogenesis, pre-spawning, spawning, and spent (post-spawning) of the female gonad development (Wildhaber et al. 2007). In b, the baseline relationship is based on data reported in Wildhaber et al. (2007).

**Reference**

Wildhaber, M.L., Papoulias, D.M., DeLonay, A.J., Tillitt, D.E., Bryan, J.L. & Annis, M.L. (2007) Physical and hormonal examination of Missouri River shovelnose sturgeon reproductive stage: a reference guide. *Journal of Applied Ichthyology,* **23,** 382-401.
