# Supplementary material for: Divergent density feedback control of migratory predator recovery following sex‐biased perturbations
Source: Ecol Evol. 2020 Apr 8;10(9):3954–67. doi: 10.1002/ece3.6153 (PMC7244814; doi:10.1002/ece3.6153)
Supplement: Supplementary file 4 — Figure S3 [file ECE3-10-3954-s004.docx]

**Figure S3**. Temporal patterns of demographic and reproductive traits of simulated shovelnose sturgeon populations under the baseline scenario – a) adult sex ratio, b) female number, c) relative female gonad mass, d) fecundity, e) spawner biomass, f) recruit number, and g) a stock-recruit relation. The sturgeon model used for the baseline simulation has been calibrated and validated with field survey data on the lower Platte River population ([Goto *et al.* 2015](#_ENREF_21)).

**Reference**

Goto, D., Hamel, M.J., Hammen, J.J., Rugg, M.L., Pegg, M.A. & Forbes, V.E. (2015) Spatiotemporal variation in flow-dependent recruitment of long-lived riverine fish: Model development and evaluation. *Ecological modelling,* **296,** 79-92.
